# Prevalence and associated factors of resting electrocardiogram abnormalities among systemic lupus erythematosus patients without cardiovascular disease

**DOI:** 10.1186/s13075-017-1240-1

**Published:** 2017-02-10

**Authors:** Hanan Al Rayes, Paula J. Harvey, Dafna D. Gladman, Jiandong Su, Arthy Sabapathy, Murray B. Urowitz, Zahi Touma

**Affiliations:** 1 Lupus Clinic, Centre for Prognosis Studies in the Rheumatic Diseases, Toronto Western Hospital, University of Toronto, EW, 1-412, 399 Bathurst Street, Toronto, Ontario M5T 2S8 Canada; 2 Women’s College Research Institute, Women’s College Hospital, University of Toronto, Toronto, Ontario Canada; 3 Toronto Western Research Institute, University of Toronto, Toronto, Ontario Canada

**Keywords:** Cardiovascular disease, Systemic lupus erythematosus, Electrocardiogram

## Abstract

**Background:**

Electrocardiogram (ECG) cardiovascular disease (CVD) abnormalities (ECG-CVD) are predictive of subsequent CVD events in the general population. Systemic lupus erythematosus (SLE) patients are vulnerable to CVD. We aimed to determine the prevalence of ECG-CVD in SLE patients and to examine the risk factors associated with ECG-CVD.

**Methods:**

A 12-lead resting supine ECG was performed on consecutive adult patients attending the clinic. One cardiologist interpreted the ECGs. ECG-CVD were defined as the presence of one or more of the following 4 elements (ECG-4): ST-segment and/or T-wave abnormalities, left ventricular hypertrophy (LVH), left axis deviation (LAD), left bundle branch block (LBBB) and right bundle branch block (RBBB). ECG-5 included the same elements as ECG-4 and the Q-wave. Repeated measurement data were created and the associations between ECG-4/ECG-5 and demographics were evaluated with univariate and multivariate Cox regression models.

**Results:**

Of 487 SLE patients, 104 (21.4%) and 118 (24.2%) patients had one or more of the ECG-4 and ECG-5 elements, respectively. A higher prevalence of ECG-CVD was found in patients with a longer SLE disease duration, and the burden of ECG-CVD elements increased with age. Increased age, active SLE disease, and damage were associated with ECG4 and ECG-5, while treatment of hyperlipidemia was protective.

**Conclusion:**

A high prevalence of ECG-4 (21.4%) and ECG-5 (24.2%) was found in this SLE cohort. Controlling SLE disease activity is important since it was associated with ECG-4 and ECG-5. Early identification of ECG-4 and ECG-5 in SLE patients might allow for better stratification and risk management.

## Background

Substantial progress has been made in the awareness and prevention of coronary artery disease (CAD) in patients with systemic lupus erythematosus (SLE) since the first published reports in long-term observational cohort studies of clinical CAD in an estimated 6–10% of SLE patients [1, 2]. The prevalence of myocardial infarction (MI)/angina and sudden death in the Toronto SLE clinic was approximately 10% in 1995 [3], and a similar prevalence of CAD has been reported in other SLE cohorts [4, 5]. SLE is now recognized as an independent risk factor for cardiovascular disease (CVD) [6] and, as such, was incorporated into the recently revised American Heart Association guidelines for the prevention of CVD in women [7]. Abnormalities detected on resting electrocardiogram (ECG) in healthy adults are associated with an increased risk for subsequent CVD events [8]. In a systematic review, Chou et al. [8] found that resting ECG abnormalities (ECG-CVD 4-elements), in particular ST-segment and/or T-wave abnormalities, left ventricular hypertrophy (LVH), left axis deviation (LAD), left bundle branch block (LBBB) and right bundle branch block (RBBB), were associated with subsequent CVD events (e.g., sudden coronary heart disease death, nonfatal myocardial infarction, and congestive heart failure). In their systematic review, Chou et al. reported other resting ECG abnormalities (e.g., prolonged QT interval, Q waves, arrhythmia, and others) but these ECG findings were evaluated by too few studies or were too variably defined to have clear conclusions about their usefulness as predictors of subsequent CVD events [8]. The objective of this study was to determine the prevalence of ECG-CVD (4-elements (ECG-4) as per Chou et al., and 5-elements (ECG-5) as per Chou et al. or Q wave) in a cohort of SLE patients, and to examine the factors associated with ECG-4 and ECG-5.

## Methods

### Setting

A standard digitally recorded 12-lead resting supine ECG was performed on consecutive adult SLE patients ≥18 years, with 4 or more of the American College of Rheumatology (ACR) criteria or 3 ACR criteria plus a typical histological lesion of SLE on renal or skin biopsy [9] attending the University of Toronto SLE Clinic from October 2011 to November 2015. All patients were evaluated according to the standard clinic protocol which includes demographics, assessment of disease activity (SLE Disease Activity Index 2000 (SLEDAI-2 K)) [10] and damage (SLICC/American College of Rheumatology Damage Index (SDI)) [11], current and past glucocorticoid, antimalarial, antihypertensive, and immunosuppressant medication use, and laboratory tests at each visit. Patients were followed prospectively every 2 to 6 months. The following data were collected on all patients as part of the regular databank protocol and were made available for this study. Patients were considered to have a positive smoking status if they were documented as a current smoker at any point in the 5 years preceding the date of ECG. History of cardiovascular events (MI, angina, angioplasty, congestive heart failure and pacemaker), diabetes mellitus (DM) 3 years before ECG, hyperlipidemia on statins ever prior to ECG, and hypertension within 5 years from ECG (>140 mmHg systolic or >90 diastolic mmHg, or using medication for hypertension) were extracted. Patients were considered positive for antiphospholipid antibodies (aPLA) if they had ever been positive for aPLA, including total anticardiolipin antibodies and individual anticardiolipin antibodies (aCL IgG/aCL IgM) (Phadia Varelisa kits, Somagen for anticardiolipin antibodies screen and IgG and IgM assays) measured yearly by enzyme-linked immunosorbent assay (ELISA) [12]. The clotting assays for SLE anticoagulant included the dilute Russell viper venom time and the platelet neutralization procedure. SLE anticoagulant (LA) was considered present if patients tested positive on two or more occasions since joining the SLE cohort. Other laboratory variables were evaluated: ANA, anti-ds DNA, anti-Ro, anti-La, anti-Smith, anti-RNP, anti-Jo1 antibodies, ANCA, and Coombs tests (ever-positive 5 years prior to ECG).

The ECGs had all identifying data removed and so were read “blind”. The coded 12-lead ECGs were evaluated and interpreted by one senior cardiologist (PH) using the Minnesota code classification system, which is a widely used method of categorizing ECG abnormalities [13].

All patients provided informed consent for the collection, storage, and use of clinical and laboratory data following procedures in accordance with the Declaration of Helsinki and approved by the University Health Network Research Ethics Board, Toronto, Ontario, Canada.

### Patient selection

Consecutive SLE patients seen at the SLE clinic from October 2011 to November 2015 were recruited to this study. Patients were excluded if they had had a CVD event (MI, angina, congestive heart failure (CHF), angioplasty and pacemaker) prior to ECG.

### Study design

**Fig. 1:**
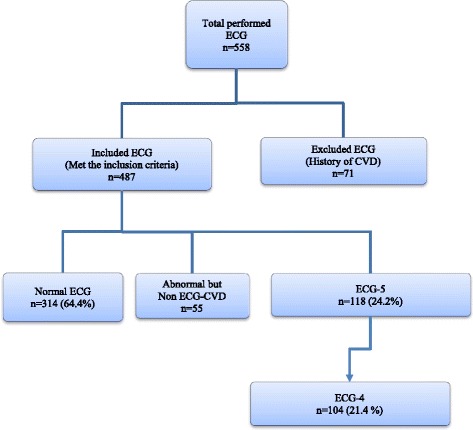
Study design and grouping of patients: normal electrocardiogram (*ECG*), non-ECG-cardiovascular disease (*ECG-CVD*), ECG-4, and ECG-5. Non-ECG-CVD defined as the presence of any of the following ECG abnormalities: right axis deviation, arrhythmia, sinus tachycardia, sinus bradycardia, atrioventricular blocks, atrial ectopic rhythm, and atrial enlargement

SLE patients were grouped into those with normal ECG and those with ECG-CVD. ECG-CVD patients were further grouped into two: ECG-4 (with one or more of the following abnormalities—LBBB/RBBB, LAD/left anterior fascicular block (LAFB), LVH, and ST-segment and/or T-wave abnormalities); and ECG-5 (any of the above or pathological Q wave) (Fig. 1). Our study focuses on patients with ECG-4 and ECG-5 compared to patients with a normal ECG. Descriptive analyses of patient demographics, disease activity, damage, and antibody abnormalities were studied in the following groups: patients with normal ECG, patients with ECG-4, and patients with ECG-5. The clinical/laboratory data available at the time of ECG were used to study the association with ECG-CVD. The adjusted mean SLEDAI-2 K (AMS) was calculated using SLEDAI-2 K 2 years prior to the ECG visit [14].

### Statistical analyses

Simple statistics at baseline (at the first visit to the clinic) were described by mean and percentages for continuous and categorical variables. The differences between the group of patients with normal ECG and the group of patients with ECG-CVD (ECG-4 and ECG-5) were compared by *t* test and chi-square test.

A counting process data structure was created for the outcome of both ECG-4 and ECG-5. Patient sex, ethnicity, time from first visit to the clinic to the time of ECG, hypertension, hyperlipidemia on statin therapy, laboratory test (antibodies), smoking ever before ECG, disease activity, and diabetes 3 years before ECG test were extracted as time-fixed variables. Other variables such as organ damage and treatment were extracted as time-variant variables.

Univariate Cox regression was performed to test each risk factor, comparing normal ECG and ECG-4 and ECG-5. The variables with *p* value less than 0.2 were entered into the multivariate regression. The traditional risk factors for CAD including hypertension and smoking were forced into the model.

Cox regression proportionalities were checked by the Schoenfeld residuals, and continuous variables were tested for their linearity using Martingale residuals. The time-dependent multivariate Cox regression model was built using a step-down covariate-selection approach; Akaike information criterion (AIC) adjusting the number of covariates in the model was adopted as the method assessing the goodness of fit. Both hypertension and smoking were forcedly added to the multivariable regression but did not show an association with ECG-4 and ECG-5. The analysis was conducted using SAS (9.3) and *p* values <0.05 were considered statistically significant.

## Results

### ECG

**Fig. 2:**
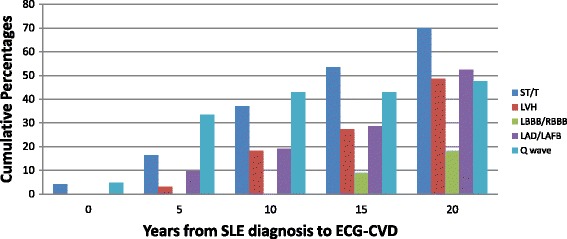
Cumulative proportions of individual ECG-5 from systemic lupus erythematosus (*SLE*) diagnosis up to 20 years of follow-up (e.g., 11 out of 21 (52.4%) patients had LAD/LAFB up to 20 years and the other 10 LAD/LAFB occurred after 20 years of follow-up). *ECG-CVD* electrocardiogram cardiovascular disease abnormalities, *LAD* left axis deviation, *LAFB* left anterior fascicular block, *LBBB* left bundle branch block, *LVH* left ventricular hypertrophy, *RBBB* right bundle branch block

Among the 558 adult SLE patients who had an ECG, 44% were inception (patients who joined the SLE clinic within 1 year from the diagnosis of SLE). Seventy-one patients were excluded as they had CAD before the ECG test. A total of 487 ECGs were evaluated of which 314 patients (64.4%) had a normal ECG. The prevalence of ECG-4 was 21.4% (104/487 patients) and the prevalence of ECG-5 was 24.2% (118/487 patients). The most common element of ECG-4 was ST-segment abnormalities and/or T-wave abnormalities (73/104; 70%), followed by LVH in 32%, LAD or LAFB in 20%, and LBBB or RBBB in 11%. In ECG-5, Q-wave was found in 18% (21/118) of the patients. The ECG-CVD elements were found as one ECG-CVD element in 67% (70/104) in the ECG-4, two ECG-CVD elements in 25% (26/104), and 7% (7/104) had three ECG-CVD elements. ECG-CVD was seen more often with longer disease duration (Fig. 2). Other ECG abnormalities documented in 55 patients were: right axis deviation in 9 patients, incomplete RBBB in 3 patients, atrial enlargement in 7 patients, and arrhythmia in 36 patients (sinus tachycardia in 9 patients, sinus bradycardia in 11, short PR in 8, first A-V block in 3, ectopic atrial rhythm in 3, premature atrial contraction in 3, and ventricular bigeminy in 1 (few patients had overlaping types of arrhythmia).

### Patient characteristics

The baseline (at first visit to the clinic) characteristics of the patients were similar in SLE patients with a normal ECG and those with ECG-CVD (Table 1). A higher prevalence of ECG-4 and ECG-5 was identified among older SLE patients. Patients with longer SLE disease duration had more prevalence of ECG-4 and ECG-5 compared to patients with a normal ECG (19.48 ± 11.03 vs. 15.23 ± 10.16, *p* < 0.001). Patient with longer lupus follow-up duration (13.69 ± 11.29 years) at the time of ECG had a higher prevalence of ECG-4 (*p* = 0.006) versus a normal ECG with a follow-up duration at the time of the ECG of 10.57 ± 9.40 years. Also, patients with longer lupus follow-up duration at the time of ECG have a higher prevalence of ECG-5. Hypertension within 5 years prior to the ECG was associated with a high prevalence of ECG-4 and ECG-5 (*p* = 0.015 and *p* = 0.006, respectively). There were no significant differences between a normal ECG and ECG-4 or ECG-5 among the other variables (SDI, SLEDAI-2 K, smoking, DM, and treatment with antimalarials or immunosuppressives) (Table 1). There was no difference in the antibodies profile between normal ECG and ECG-4 and ECG-5 (data not shown).

**Table 1:** Demographic and clinical characteristics of SLE patients with a normal ECG and ECG-4 and ECG-5 at the first visit

Variables		ECG normal n = 314	ECG-4 n = 104	*p*	ECG-5 n = 118	*p*
Gender						
	Female	286 (91.1%)	92(88.5)	0.43	104 (88.1%)	0.357
	Male	28 (8.9%)	12 (11.5%)		14 (11.9%)	
Ethnicity						
	Caucasian	181 (58.2%)	59 (57.8%)	0.09	69 (59.5%)	0.146
	Black	51 (16.4%)	26 (25.5%)		27 (23.3%)	
	Asian	38 (12.2%)	10 (9.8%)		12 (10.3%)	
	Others	41 (13.2%)	7 (6.4)		8 (6.9%)	
Age at SLE diagnosis (years)		29.6 ± 11.3	30.66 ± 11.01	0.42	30.46 ± 11.86	0.50
Age at ECG (years)		44.87 ± 12.9	50.14 ± 14.1	<0.001	49.95 ± 15.01	<0.001
Disease duration at ECG (years)		15.23 ± 10.2	19.48 ± 11.03	<0.001	19.49 ± 11.44	<0.001
Follow-up duration at ECG (years)		10.57 ± 9.4	13.69 ± 11.29	0.006	13.41 ± 11.24	0.008
SLEDAI-2 K at the first visit		9.24 ± 8.3	10.2 ± 7.78	0.30	9.82 ± 7.67	0.52
SDI		0.27 ± 0.7	0.44 ± 0.92	0.07	0.44 ± 0.90	0.06
Cumulative glucocorticoid (g)		5.24 ± 16.5	9.16 ± 31.25	0.10	8.96 ± 29.85	0.10
Treated with antimalarials		147 (46.8%)	51 (49.0%)	0.69	59 (50.0%)	0.55
Treated with immunosuppressives		93 (29.6%)	33 (31.7%)	0.68	37 (31.4%)	0.73
Hypertension		153 (48.7%)	65 (62.5%)	0.015	74 (63.6%)	0.006
Hyperlipidemia on statins ever before ECG		87 (27.7%)	40 (38.5%)	0.039	44(37.3%)	0.05
DM 3 years prior to ECG		16 (5.1%)	4 (3.8%)	0.60	4 (3.4%)	0.45
Smoking ever prior to ECG		91 (29%)	30 (28.8%)	0.97	34 (28.8%)	0.91

Values are shown as mean ± SD or *n* (%) as appropriate

*p* values are from *t* test for means, Chi-Square test for binary variables, and Cochran-Armitage trend test for categorical variables

*DM* diabetes mellitus, *ECG* electrocardiogram, *SDI* SLICC/American College of Rheumatology Damage Index, *SLE* systemic lupus erythematosus, *SLEDAI-2 K* SLE Disease Activity Index 2000

### Univariate analysis

Both ECG-4 and ECG-5 were significantly associated with non-Caucasian ethnicity, longer lupus disease duration, and with increasing age of the SLE patients at each visit. Patients with ECG-4 and ECG-5 had more active SLE disease activity (AMS 2 years prior to ECG visit) and more damage (SDI) compared to patients with normal ECG. Treatment, including glucocorticoids, antimalarials, and immunosuppressives, was associated with ECG-4 and ECG-5. Other classic CVD risk factors such as smoking and hypertension were not associated with ECG-4 or ECG-5 in the univariate analysis (Table 2).

**Table 2:** Univariate Cox regression analysis for ECG-4 and ECG-5

	ECG-4		ECG-5	
Variables	HR (95% CI)	*p*	HR (95% CI)	*p*
Non-Caucasian	1.91 (1.27–2.86)	0.002	1.63 (1.11–2.37)	0.012
Gender (male)	1.13 (0.62–2.07)	0.68	1.19 (0.68–2.09)	0.53
Age	1.03 (1.007–1.05)	0.01	1.02 (1.004–1.05)	0.02
Disease duration	1.06 (1.03–1.09)	0.0007	1.05 (1.014–1.09)	0.006
Hypertension within 5 years prior to ECG	0.88 (0.49–1.57)	0.67	0.91 (0.52–1.58)	0.73
Hyperlipidemia on statins ever before ECG	0.59 (0.30–1.13)	0.11	0.54 (0.29–1.01)	0.05
Ever smoked before ECG	0.72 (0.39–1.35)	0.31	0.75 (0.42–1.35)	0.34
Diabetes 3 years before ECG	0.75 (0.27–2.04)	0.09	0.75 (0.27–2.048)	0.57
SLEDAI-2 K	1.05 (1.012–1.013)	0.01	1.05 (1.01–1.09)	0.008
AMS 2 years prior to ECG	1.08 (1.02–1.13)	0.005	1.08 (1.03–1.13)	0.003
SDI	1.23 (1.07–1.41)	0.003	1.21 (1.06–1.39)	0.006
Cumulative glucocorticoids	1.007 (1.001–1.01)	0.02	1.007 (1.001.013)	0.02
Treated with antimalarials	2.27 (1.12–4.59)	0.02	2.11 (1.11–4.04)	0.02
Treated with immunosuppressives	2.15 (1.21–3.83)	0.009	2.06 (1.19–3.55)	0.009
Coombs	1.19 (0.81–1.76)	0.37	1.19 (0.83–1.73)	0.33
Anti-Jo1	0.65 (0.26–1.63)	0.36	0.71 (0.30–1.64)	0.41
Anti-La	0.79 (0.47–1.34)	0.39	0.8 (0.49–1.31)	0.38
Anti-RNP	1.18 (0.791.74)	0.41	1.24 (0.86–1.79)	0.24
Anti-Ro	0.81 (0.56–1.18)	0.27	0.79 (0.55–1.14)	0.21
Anti-Scl70	0.49 (0.15–1.59)	0.24	0.45 (0.14–1.44)	0.19
Anti-Smith	1.43 (0.89–2.27)	0.13	1.44 (0.93–2.22)	0.11
ANCA	0.63 (0.32–1.21)	0.17	0.63 (0.34–1.18)	0.15
Anti-ds DNA	1.52 (1.02–2.29)	0.041	1.48 (1.01–2.16)	0.04
ANA	0.86 (0.38–1.97)	0.72	0.74 (0.36–1.53)	0.42
APLA	0.85 (0.53–1.35)	0.48	0.73 (0.49–1.23)	0.29

*AMS* adjusted mean SLEDAI-2 K, *CI* confidence interval, *ECG* electrocardiogram, *HR* hazard ratio, *SDI* SLICC/American College of Rheumatology Damage Index, *SLE* systemic lupus erythematosus, *SLEDAI-2 K* SLE Disease Activity Index 2000

### Multivariate analysis

#### ECG-4

The multivariate Cox regression showed that older age, SLE disease activity (AMS 2 years prior to ECG), and SDI are associated with ECG-4. Older patients were more likely to have ECG-4, with a 4% increase in hazard ratio (HR) for every 1-year increase in age (HR = 1.05; 95% confidence interval (CI): 1.01–1.07; *p* = 0.002). Patients with active SLE (AMS 2 years prior to ECG visit) and patients with more damage (SDI) were more likely to have ECG-4 (HR = 1.08; 95% CI: 1.02–1.16; *p* = 0.009 and HR = 1.29; 95% CI: 1.1–1.53; *p* = 0.002, respectively).

Statin treatment for hyperlipidemia was protective against ECG-4 (HR = 0.43; 95% CI: 0.21–0.89; *p* = 0.02). There was a trend for immunosuppressive therapy to be associated with ECG-4 (*p* = 0.05). This trend of association can be explained by the use of immunosuppressive therapy in the context of active lupus disease, especially that ECG-4 was also associated with AMS.

#### ECG-5

The results of the multivariate analysis for ECG-5 were similar to ECG-4, and showed an association with older age (HR = 1.04; 95% CI: 1.007–1.06; *p* = 0.01), SLE disease activity (AMS 2 years prior to ECG; HR = 1.07 95% CI: 1.002–1.14; *p* = 0.04), and SDI (HR = 1.28; 95% CI: 1.08–1.51; *p* = 0.004) (Table 3).

**Table 3:** Multivariate Cox regression analysis for ECG-4 and ECG-5

	ECG-4		ECG-5	
Variables	HR (95% CI)	*p*	HR (95% CI)	*p*
Age at each visit	1.05 (1.01–1.07)	0.002	1.04 (1.007–1.06)	0.01
AMS (2 years prior to ECG)	1.08 (1.02–1.16)	0.009	1.07 (1.002–1.14)	0.04
SDI	1.29 (1.10–1.53)	0.002	1.28 (1.08–1.51)	0.004
Hyperlipidemia on statins ever before ECG	0.44 (0.21–0.89)	0.02	0.44 (0.22–0.87)	0.02
Hypertension	0.66 (0.34–1.26)	0.21	0.75 (0.4–1.39)	0.36
Ever smoked before ECG	1.13 (0.59–2.16)	0.71	1.09 (0.58–2.06)	0.77
Immunosuppressive treatment at each visit	1.88 (1.01–3.51)	0.05	1.67 (0.93–3.04)	0.09
Antimalarial treatment at each visit	1.76 (0.87–3.58)	0.12	1.81 (0.92–3.56)	0.12

Akaike information criterion (AIC) = 334.6

*AMS* adjusted mean SLEDAI-2 K, *CI* confidence interval, *ECG* electrocardiogram, *HR* hazard ratio, *SDI* SLICC/American College of Rheumatology Damage Index, *SLE* systemic lupus erythematosus, *SLEDAI-2 K* SLE Disease Activity Index 2000

## Discussion

It is well established that SLE patients have increased mortality secondary to cardiovascular events even in the absence of traditional risk factors for CVD [15]. In this study of SLE patients, the prevalence of the ECG-4, which have been shown by Chou et al. to be predictive of CVD [14], is 21.4% and the prevalence of ECG-5 is 24.2%. The prevalence of ECG-CVD among SLE patients is greater than the general population (prevalence 3.6–17%) as shown in Table 4. Chou et al. showed that ECG-4 predicts subsequent CVD events [16] and that pathological Q-wave was also a predictor of future CVD [14]. In this study, ECG-CVD (ECG-4 and ECG-5) was more common in patients aged 50.14 ± 14.1 years compared to the group without ECG-CVD (age 44.8 ± 12.9 years); however, this is still younger than the general population.

**Table 4:** Prevalence of ECG-CVD in the general population

Study	References	Sample size	Mean age (range) in years	ECG-CVD elements in different studies	Prevalence (%)
Chicago Heart Association detection project	Liao et al., 1988 [34]	17,633	51 (40–64)	• ST-segment depression • T-wave inversion • LVH • RBBB/LBBB • Complete or second AV block	11.1% (female 12.5%, male 9.6%)
Charleston Heart Study	Sutherland et al., 1993 [35]	933	48 (35–74)	• ST-segment depression • T-wave changes • LBBB or RBBB • LAD • LVH	9%
Fine Study	Menotti and Seccareccia, 1997 [16]	1785	Not reported (65–84)	• Q-QS abnormalities • ST-T abnormalities • High R. waves • Major arrhythmias and blocks	8%
Belgian Inter-University Research	De Becquer et al., 1998 [27]	9954	48 (25–74)	• ST-segment depression • T-wave inversion • LBBB or RBBB • Atrial fibrillation or flutter	3.6%
Copenhagen ECG Study	Rasmussen et al., 2014 [36]	285,194	65	• ST-segment depression	Female 7% Male 8%

The definition of ECG-CVD varied among the included studies in Table 4

*ECG-CVD* electrocardiogram cardiovascular disease abnormalities, *LAD* left axis deviation, *LBBB* left bundle branch block, *LVH* left ventricular hypertrophy, *RBBB* right bundle branch block

In our analysis, we found an association between ECG-CVD (ECG-4 and ECG-5) and lupus disease activity (AMS 2 years prior to ECG) and damage (SDI). Some studies have found an association of azathioprine with atherosclerosis [17, 18]. Although there was a trend in our study for an association with immunosuppressive drugs (azathioprine, mycophenolate mofetil, cyclophosphamide, cyclosporine, and methotrexate), this was not statistically significant. Cumulative glucocorticoid therapy has been postulated as a potential risk factor for atherosclerosis in patients with SLE [2, 19, 20], but not all studies have confirmed this association [21]. In this study, cumulative glucocorticoid therapy and antimalarial drugs were significantly associated with ECG-4 and ECG-5 in the univariate analysis, but did not remain in the multivariate analysis. Statin therapy ever for hyperlipidemia was protective against ECG-CVD in the multivariate analysis.

While some previous studies showed an association between antiphospholipid antibodies and clinical CVD and/or events [20, 22], other studies did not [17, 21, 23]. In the present study, antiphospholipid antibodies were not associated with ECG-4 or ECG-5. More recently, Andrade et al. showed that patients with antiphospholipid syndrome do not develop premature atherosclerosis [24].

Smoking, hypertension, and DM, all recognized as traditional risk factors for CVD in SLE patients [2, 23], were not significantly associated with ECG-CVD this study. Several factors could explain the lack of association with traditional risk factors for CVD in our study. First, smoking ever was reported in 29% of each of the non-CVD and ECG-4 and -5 groups, and other studies have demonstrated the importance of studying the dose-effect (pack-years) of smoking [25]. Second, in CAD, the ECG is the reflection of damage to the myocardium that usually occurs at the end of the process of the development of CAD and its clinical implications. Third, with respect to hypertension, not only is LVH a reflection of long-term inadequate control of blood pressure, but ECG has a poor sensitivity for LVH (slightly lower in females than in males). Fourth, the number of patients with DM was small (only four patients had DM in ECG-4 and -5).

In the current study, the most common element of ECG-4 was ST-segment abnormalities and/or T-wave abnormalities (70%), followed by LVH in 32%, LAD or LAFB in 20%, and LBBB or RBBB in 11%. In ECG-5, Q-wave was found in 18% of the patients. The pooled adjusted HR (for CVD) of ST-segment abnormalities in resting ECG in the normal healthy population from several studies was found to be 1.9 [26–30]. De Bacquer et al. showed that ST‐depression is predictive of CVD [31]. In a recent systematic review, both LVH LAD on resting ECG were associated with a similar increased risk for subsequent CVD events, with a pooled adjusted HR of 1.6 (95% CI, 1.4 to 1.8) [8, 16, 29–32]. LBBB was found in 3% of ECG-CVD, and it has been previously reported that, in the presence of LBBB on resting ECG, there is an increased incidence of ischemic heart disease events and/or death due to cardiovascular disease compared to control subjects [8, 33]. Interestingly, pathological Q-waves were noted in 11% of the patients with ECG-5 without a previous history of ischemic heart disease.

A limitation of this study is the lack of a control group to better evaluate the prevalence of CVD-ECG-4 abnormalities in patients without SLE. In order to evaluate the prevalence of CVD-ECG-4 abnormalities, a baseline ECG should have been done and this is also a limitation of this study. In addition, the low level of correlation among variables in the multivariate analyses is a limitation of this study; although we avoided using variables with moderate to high correlation in the multivariate analyses, some variables were inter-related to each other at a low level, e.g., older age and hyperlipidemia (correlation coefficient = 0.30), and age and dsDNA antibodies (correlation coefficient = –0.22).

## Conclusions

SLE is an independent risk factor for cardiovascular disease [17] and therefore early identification of those SLE patients at increased risk for premature cardiovascular disease is crucial to the development and implementation of effective prevention strategies in this population. The high prevalence of ECG-4 and ECG-5 in this study of SLE patients without documented cardiovascular disease suggests that baseline (and possibly follow-up sequential) ECG may be a useful non-invasive SLE screening tool for evaluation and identification of SLE patients at increased risk for cardiovascular events with relatively minimal cost burden. Prospective follow-up studies of SLE patients with ECG-4 and ECG-5 will be helpful to determine the validity of implementation of targeted cardiovascular prevention strategies in patients with ECG-CVD.

## Abbreviations

ACR: American College of Rheumatology
AIC: Akaike information criterion
AMS: Adjusted mean SLEDAI-2 K
aPLA: Antiphospholipid antibodies
CAD: Coronary artery disease
CI: Confidence interval
CVD: Cardiovascular disease
DM: Diabetes mellitus
ECG: Electrocardiogram
ECG-CVD: Electrocardiogram cardiovascular disease abnormalities
HR: Hazard ratio
LAD: Left axis deviation
LAFB: Left anterior fascicular block
LBBB: Left bundle branch block
LVH: Left ventricular hypertrophy
MI: Myocardial infarction
RBBB: Right bundle branch block
SDI: SLICC/American College of Rheumatology Damage Index
SLE: Systemic lupus erythematosus
SLEDAI-2 K: SLE Disease Activity Index 2000
